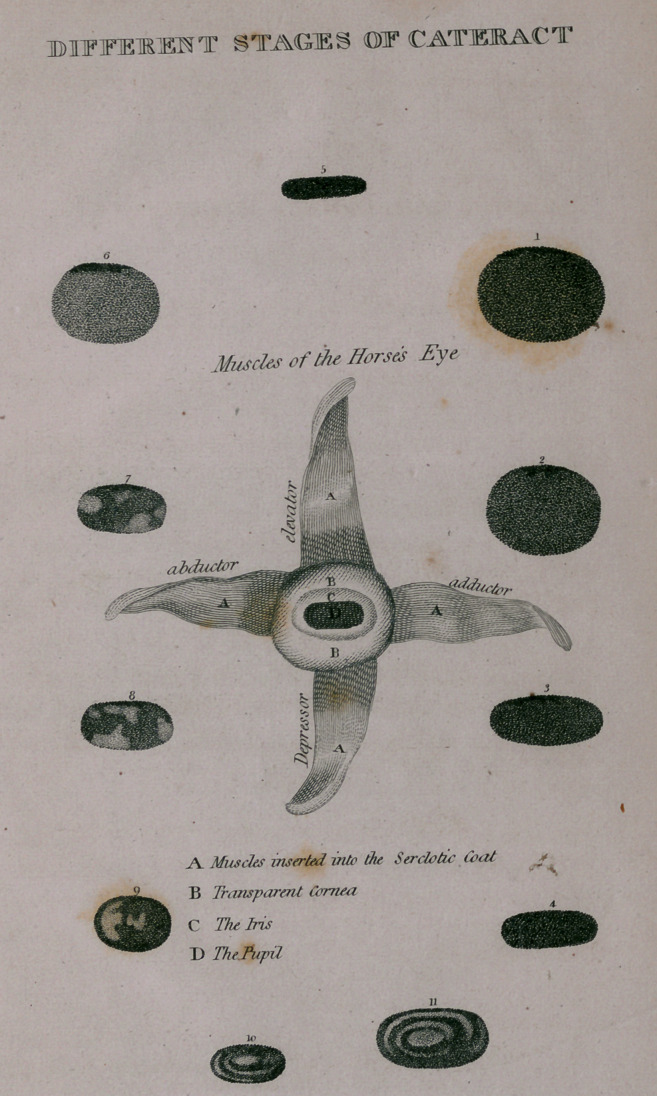# Pathology of the Horse’s Eye

**Published:** 1818

**Authors:** James Carver

**Affiliations:** V. S.


### VETERINARY OBSERVATIONS.

### PATHOLOGY OF THE HORSE'S EYE;

### BEING A DEMONSTRATION OF ITS DISEASES, CAUSES, SYMPTOMS, AND BEST MODE OF TREATMENT.

THE organ of vision, with its phenomena, have engaged the attention of the curious in every age. As a subject of admiration, it is certainly worthy of our attention; and as a subject of importance to the well being of the animal, it is even more so, as a matter which ought to command a great portion of the research of the veterinary physician and surgeon, since it is an organ very liable to disease, and of such a nature as to have hitherto baffled most of our attempts to relieve them. It is very remarkable, that though the diseases of the human eye are estimated at more than a hundred, yet there is only *one very common* to the horse; but which in obstinacy and ill effects, more than equals all the human catalogue. This is ophthalmia membranum, or inflammation of the coats of the eye and its consequences, which by common farriers is termed moon blindness. The ophthalmia tarsi of man finds no place in the diseases of the horse.

This disease (ophthalmia) is, as I shall show, a specific and constitutional inflammation of the eye, and as such requires, probably a complete alteration of, in the constitution to its cure. And although my worthy preceptor, Mr. Coleman, has paid more attention to this subject, perhaps, than any other veterinarian in Europe, or perhaps in the world, and yet the result of his researches has unfortunately been to confirm the character of its obstinacy, and of its *fatality* to the organ it affects.

It were much to be wished that the diseases to which the eye of the horse is subject, were as much under the control of our art as some of those of the feet; but when we look at the number of blind horses, and compare them with those that are permanently lame, the difference will be manifest. It is now admitted by all veterinarians, that there is a greater disposition to disease in the horse's eye than of any other animal we domesticate; yet the horse is treated in a manner far more artificial (I should be warranted in saying more *preposterously*) than most other animals; we ought not, therefore, to wonder at meeting more frequently with instances of disease in the eye, particularly when the delicacy of the structure of that organ is considered. It is, however, of importance to discriminate between those diseases of the eyes which are produced by dust, straws, insects, blows, or any external mechanical cause, and such as are brought on by original defects in that organ, *aided* by the *slow*, gradual, and imperceptible effects of the hot and foul air of stables; because the former will be found to yield to the usual remedies that are employed for general inflammation, provided they are used with diligence and judgment. For which reason, on the first appearance of inflammation, it will be prudent to examine the inside of the eyelid with caution, and if any insect or mote be discovered, it should be carefully removed. In all cases where the lids are much swelled, it is good practice to scarify the inner membrane freely with a small lancet, and to foment them for ten or fifteen minutes with a weak infusion of camomile flowers, or warm milk and water, in order to encourage the bleeding. If the inflammation be very great, a veterinary practitioner should be called in, the pulse examined as to the necessity of bleeding, and according to circumstances, the quantity of blood to be drawn away.

Opening the angular veins under the eye I have sometimes found of great advantage, and the fomentations should be continued, and at night the inflamed organ should be covered with a large poultice of bread and milk and a little sweet oil, to prevent it proving hard.* Puncturing the transparent cornea to let out the aqueous humour is sometimes attended with very beneficial effects. There is no danger attending this operation, as the humour secretes again in twenty-four hours. The horse at the same time should be *marshed*, and a mild dose of physic administered, if necessary. In case the stable the horse stands in should be hot and low, he should be removed to one that is lofty and airy, and if the inflamed eye affected be extremely susceptible of the stimulus of light, which may be known by the horse keeping it constantly closed, it will be of use to darken the stable, or at least to shut out the strong light of the sun. But darkness, however desirable in such cases, is certainly not worth purchasing at the expense of cool pure air. As soon as the violence of the symptoms begin to abate, and the horse opens his eye pretty freely, a little cooling eye-water may be applied two or three times a day with the corner of a soft sponge. But if the inflammation of the inner coat, called the retina, be extremely violent, which is known by the animal keeping his eye closed, the bleeding, scarification, and physic must be repeated at the end of five or six days. Blistering is always attended with general good effects, but from the corrosive materials with which they are generally compounded by those who *assume* a knowledge of veterinary medicine, they generally leave a scar for life; but if properly mixed are far preferable to seaton or rowel in the neighbourhood of the diseased organ. For whilst the practice must be allowed to be at any rate more cleanly, and free from fetor arising from rowels and seatons, I have always found it attended with effects equally beneficial, more especially as the blistering may be repeated from time to time, as soon as the scab falls off from the skin,

* A large roasted apple applied moderately warm to the inflamed eye, I have commonly found of great advantage; you may take out the core of the apple, and beat up the pulp with molasses and honey, if you please—so much the better.

without any fear of blemishing the cheek, provided only that judicious materials be used for the blister. Gentle exercise should be had recourse to for two hours every day; but if in summer always in the shade.

When the violence of the inflammation has abated, the animal should be turned to grass for two or three hours every day; but if in summer always in the shade.

When the violence of the inflammation has abated, the animal should be turned to grass for two or three hours every day; for there can be no question that the disadvantage of holding down his head to graze, will be more than counterbalanced to the animal by the good that will result from the effects of the green food upon the bowels, and more especially by the application *of the fresh cool air to the eye.*

This mode of treatment, during my stay on Long Island, I generally found effectual in all inflammations which were brought on by any external mechanical cause, and it is in fact all that can be done even when hot and foul air has produced the disease, by slow and imperceptible degrees. But, in the latter case, though the eye will look clear for a time, it will again become inflamed and dull, and the cornea loosing its natural transparency, will become opake, having sometimes a yellow, or muddy, at others, a dim and blue appearance. In the latter case, it is often supposed, *that a flim has grown over the eye*, and from grooms, smiths and jockeys, has received the name of *moon blindness*, from an absurd and foolish notion, like many other things, handed down from *William*, *Dick*, to *Harry*, that the moon had influence over such eyes,—whereas the moon has just as much to do with it, as I have with the pope of Rome. Now, as I know of no remedy for *cronic inflammation* of the eyes, called by those wise men *moon blindness*, which almost always ends in cataract, I think it almost useless to subscribe or recommend the trial of any application. But as such eyes do sometimes get so clear as to pass for sound ones, even with dealers, who pride themselves upon their judgment in those matters, it is always best to part with the horse: for *I do positively assert*, that no man who has not properly *studied* the anatomy, pathology, and functions of *that* organ, can, or ever will be, competent to judge of its diseases; and as the mode and conditions of parting with such a horse, comes not within my province to determine, I speak as a veterinarian, not as a *casuist.*

Many writers have laid great stress upon the use of stimulating eye waters, and powders blown into the eye* in those cases, where on account of the blueness of the cornea, (or outward coat,) a flim has been *supposed* to have grown over the eye,—but as the eye will, in many instances, partially recover its transparency, without any application, and as I have seldom seen any decided or permanently good effect for any particular application, whether mild or violent, I have, I confess, no faith in the many pretended cures which are said to be effected by them. The truth seems to be, that there is some peculiarity of structûre in the outer coat of the horse's eye, which, when it is attacked with cronic inflammation, precludes the successful application of the most powerful remedies. Nevertheless I have thought it necessary in the *incipient stage* of the disease to subjoin two formulæ for eye powders, which, in such cases, a little may be rubbed† into the eye two or three times a week, with as good prospect of success as any I have seen tried. Of times, however, a very considerable degree of blueness or dimness will remain in the outer coat of the eye, in consequence of inflammation that may have been brought on by blows, wounds, or external injuries; and it would be most prudent in those cases to refrain from any application to such specks or opaci-

* Grooms, smiths and jockeys will inform you, that glass *bottles*, *pounded fine*, is a most effectual remedy.

† As respects rubbing it into the eye, it is in the following manner: By pressing the eye lid open with one thumb, and wetting the other with the mouth, sufficient will stick to the part wetted to be rubbed into the eye.

ties, especially if they do not obstruct vision, as they will ultimately, nearly, if not wholly, be absorbed. The peculiar colour or appearance, however, of such opacities, (as *contra-distinguished*) from the opacity of moon blindness, it would be easy to describe. Commonly they are circumscribed, and they are never diffusing themselves over the whole of the outer coat of the eye. The frequency of what is called moon-blindness among horses, is indisputably, chiefly, owing to the hot and foul air of stables; but that there is an hereditary tendency to the inflammation of the eyes of some horses, which no care or prudence can guard against, no candid man, who is in possession of sufficient facts to guide his judgment, can for a moment hesitate to admit. Of late, however, it has become fashionable *to deny and decry* the existence of hereditary disease; which, to me, seems as indefensible, as it would be to deny the existence of the sun in the firmament.

The eye of the horse is provided by nature with a partial membrane, called by anatomists *membrana nictitans*, the effects of which may be seen exemplified in the day time by birds of prey, and even during strong sun-shine, by the common poultry; this is also beautifully exemplified by visiting a hen-roost at night by candle-light. Under ordinary circumstances, this membrane would seem to be but of small use to the horse, but in that fatal disease called lock jaw, its use is most beautifully exemplified, especially in the last stage of that disorder.

For if the horse be suddenly approached, in this highly irritable state of the nervous system, this membrane is instantly expanded to its full extent, (about one half of the globe) and the eye being at the same moment turned inwards, towards the nose, the pupil is thus as completely covered, and the light as effectually shut out from the retina, as if the membrane were large enough to cover the whole of the globe. Now, after a long continuance of moon blindness, this membrane becomes reddened and thickened, and the farriers who call it the (haw) or hooks, mistaking *effect for*

*cause*, imagine that this occasions the recurrence or continuance of the complaint, and therefore (acting rightly on wrong principles) they cut out the membrane, which nine times out of ten produces total blindness, and sometimes a few instances may be produced of the eye remaining sound after the haw has been cut off.

Further, I have no hesitation in adding, that the operation of cutting out the hooks, as it is called, is, in all cases, a barbarous ignorant custom, inasmuch that it robs the animal of a membrane which certainly was not made in vain, but placed by Providence in that part of the organ, as the curtain of the eye, to act as a defence against the superfluous rays of light, when the eye is offended by that, or some other foreign power.*

* On this subject, Professor Coleman and also Dr. Munro, in their lecture on Comparative Anatomy, make the following interesting remarks: All quadrupeds have, at the internal cauthers or corner of the eye, a firm strong membrane, with a cartilaginous edge tipped with black, and which in different animals is made to cover the eye, as they are more or less exposed to dangers in search of food.

This membrane nictitans, as it is called, is, however, not so large in all these animals. Cows and horses have it however so large, as to cover one half of the eye, and sometimes transparent enough to admit of some rays of light. Fishes have a cuticle to cover their eyes, as they are always in danger in that inconstant element in which they live. In this, therefore, we may observe a sort of gradation. But all quadrupeds have a seventh muscle, called by some the suspensorious. It surrounds almost the whole of the optic nerve, and its use is to sutsain the weight of the globe of the eye, and thereby prevent the optic nerve from being too much stretched, without obliging the four straight muscles to be in continual contraction, which would be inconvenient: at the same time this muscle may be brought at the pleasure of the animal to assist any of the other four, by causing one particular portion of it to act at a time.

We may also here remark, that the figure of the pupil, which is different in different animals, is always exactly adapted to the creative way of life, as well as to the different species of object that are viewed. Man has his circular, an ox has it oval, with the largest diameter placed transversely, to take in a larger view of his food. Cats have theirs also oval, but the largest diameter placed exactly the reverse perpendicular, which en-

In writing upon inflammation of the eyes, I should perhaps be considered, by my medical readers, as guilty of a very great omission, were I to refrain saying something upon

### CATARACT, the most common and universal result of moon blindness, especially as the *cataract* in the human subject is frequently treated successfully by surgeons. But it does not appear that the subject of this disease among horses has ables them to exclude a stronger light altogether, or admit that only which is necessary. This is beautifully exemplified by taking a cat from a bright sunshine light into a dark cellar, and so vice versa. The pupils of different animals also varies in wideness according as the organs of vision are more or less acute. Thus cats and owls, who seek their prey in the night and dark places, have the pupils in the day time contracted into a very small compass, as a great number of rays would oppress their nice organs; whilst in the night, or where the light is faint, they open the pupil for the admission of more rays.

The posterior part of the choride coat also, which is called the tapetum, is of different colours in different animals. Horses, oxen, sheep, deer, &c. feeding mostly on grass, have this membrane of a green colour, that it may reflect on the retina all the rays of light that come from the objects of that colour, though that of the horse varies a little in this respect, having only one half green and the other half black, which is the nigrum pigmentum, which absorbs the rays of light, gives him better vision, and causes the rays of light, when very strong, to be less offensive to the optic nerve.

Cats and owls have their tapetum, of a whitish colour, and for the same reasons have their pupil very dilatable, and their organs of vision very acute, for we find that all animals see more or less distinctly in the dark, according as their tapetum approaches nearer to a black or white colour. Thus it is that dogs who have it of a grayish colour, distinguish objects in the night better than man, whose tapetum is of a dark brown colour, and who, I believe, sees worse in the dark than any other animal. The difference then of the tapetum, as indeed the fabric of any other part in different animals, always depends on some particular advantage accruing to him in its particular manner of life for this singularity.

Now the greatest part of insects have more than two eyes. The spider and scorpion have eight, some have more than a thousand eyes collected in two orbits; and anatomists tells us, that sixteen thousand eyes have been counted in a fly; in a beetle, 64,660, and in a butterfly 64,660.

been operated upon with success, although the attempt has been made by some of the first operators in London. This want of success may be explained, in part at least, by the utter impossibility of steadying the horse's eye during the operation.

For, the horse is provided with a muscle at the back of the globe, called the retractor, which does not exist in the human eye, and with which he acts powerfully when that organ is offended. And hence arises a common observation of grooms and stablemen, in cases of high and active inflammations, that the horse's eyes seem sunk in his head, which indeed is literally the case; for when the stimulus of light becomes painful to the highly inflamed retina, the animal acting instinctively with the retractor muscle, is enabled to withdraw the eye within the socket, and thus avoid a considerable portion of that pain which he would otherwise be exposed to.

The effectual application of a speculum, therefore, is in this way precluded, and the operator's fingers alone are inadequate to the desired purpose. And, after all, both Professor Peal, as well as Professor Coleman, says, that without the aid of glasses, the operation, however successfully performed, would be useless in its effects to the animal, always producing confused vision, and causing the horse to start at every object he sees.

Having now brought together most of the material facts respecting the treatment of the common diseases of the eye of the horse, what I have to add on the subject of their prevention, will be comprised in the sentiments of some of our first professors, which will no doubt convince my readers, that the far greater number of instances of disorders of the eyes are produced by the effluvia from the dung and urine of hot stables, with those that are met with among such as are kept in cool, airy, well ventilated stables, though the diet of both be equally nutritious, we shall cease to be sceptics; and though it is commonly imagined, that high feeding is the chief cause of those frequent diseases, it has in fact but little or no concern in the affair. Much less has any slight change in the diet of the animal any thing to do with the complaint, though this is often supposed to be the case, even by many people who appear to possess *good sense and discernment in other particulars*. Thus it is, that one often hears disorders of the eyes attributed to new corn, beans, new hay, bad water, and even *the moon*, so prone are people to investigate the latent causes of diseases in horses, and still so desirous to account for every deviation from health. But, though I say thus much, I do not mean to deny that high feeding, by inducing a state of plethora, may predispose the organ to inflammation, especially in the cases of such horses as do not get regular work or exercise.

Still, however, I repeat, that the grand exciting cause of complaints in the eyes of horses, is, most unquestionably, to be sought for in the foul air of stables. Nor is this indisputable fact to be referred (as is commonly supposed) solely to the cause of heat in the air, (which indeed is notorious to every one,) but chiefly to the presence of volatile alkali, which abounds in the air of stables, and operates unceasingly, though *imperceptibly*, as a morbid stimulus to the outer coat of the eye. So that we can scarcely expect success in inflammations of this organ, from the most powerful remedies, so long as the animal remains exposed to the *original exciting* cause. And it may not be amiss to observe here, that the existence of so prodigious a quantity of *volatile alkali*, as the air of stables abounds with, was entirely overlooked, until the number of cases of diseased eyes in Woolwich and other barracks, became so serious, as to cause the inquiry of a serious investigation, as to the causes that produced so much blindness in the cavalry. Indeed so serious was the loss of cavalry horses, from various diseases originating from the same cause of ill ventilated stables, that government at last found it absolutely necessary to appoint Professor Coleman inspector of all the country barracks throughout England, Ireland and Scotland; and it was not until the year I arrived at the college, that Mr. Coleman made his first tour, with a view of effecting a general ventilation through all the cavalry barracks; and so beneficial has been the result, that more than a hundred horses in every regiment are now saved from disease by this salutary measure. And on my first arrival in this city, it was my first object to prevail on the citizens of Philadelphia to adopt this measure; and so earnest has been my arguments for its general adoption, (although against my own interest to do so,) that had I been a divine, preaching from the pulpit, praying to send my congregation to heaven, I could not more earnestly have pleaded my cause for the benefit of the animal creation. But all in vain. I will therefore drop the subject with only one more hint. Supposing every gentleman who owns horses, would take the same trouble to run a stove pipe; or a tube of any kind, through the ceiling of their stable to the roof, the same as they do in their *coffee houses*, how much disease might be prevented? A few pigeon holes at the bottom of every door, and also through the wall at every convenient place, would be all that is required to drive the effluvia through the ventilator.

To the human subject, we know the danger of all animal and vegetable matter retained and pent up, must be, in a certain degree, equally detrimental to the constitution of a horse, my comparison therefore may be summed up in a few words. Let every owner bear in mind, that he has as much right to command in the stable as in his parlour, and were these points, which I have from the best of motives only glanced at, kept more in view, and strictly observed, how few horses would be lost by what are termed stable diseases.

And as a striking proof of the fact I am insisting upon, among others which might be adduced, I will mention a fact, which before my attention was called to the investigation of this subject, puzzled me to account for. The circumstance I allude to occurs to workmen who are employed to lime wash the walls and ceilings of filthy stables, where this salutary and necessary measure is rarely, if ever, resorted to. On which occasions I have observed, that they are often obliged to desist from their work frequently, and run out into the open air to escape the pungent atmosphere of the stables, which effects both the organs of sight and smell as violently as if hartshorn had been sprinkled about the place. Now the fact is, that volatile alkali, (called in the new nomenclature ammonia,) to the presence of which in hartshorn its pungency is entirely owing, is deposited in the air in large quantities, on the ceilings and walls of all stables, but especially of such as are low and ill ventilated, in consequence of the constant putrefactive fermentation of the litter; it therefore can admit of no question, that volatile alkali, even in its mild state, is highly prejudicial to the lungs and general system of the horse, and especially so to the eye, even when that organ is in a healthy state. How much then must it be to an inflamed eye, through the medium of hot air, to exasperate a disease, which is in many cases by the slow and silent operation of this pernicious stimulus.

These facts which I have brought together, will, I hope, serve to show the correctness of my arguments, as well as show the necessity of a more strict attention to the ventilation and cleanliness of stables: for there is no fact, of the truth of which I am more perfectly convinced, than the following; namely, that with the exception of the vicissitudes of heat and cold, to which horses are perpetually exposed, there is no circumstance appertaining to their general treatment, which operates so *constantly* and *uniformly* to their disadvantage, as the volatile alkali which is diffused in the atmosphere which they breathe. Indeed, if I were permitted to argue the point at full length, and with all the circumstances connected with it, it would not be difficult to prove, that it is more inimical to horses than the vicissitudes of temperature which they are constantly called upon to endure.

And, for this plain reason, that it is usual with all thinking people to take every measure of prevention, in order to guard as much as possible against the effects of such vicissitudes. I will now conclude this subject by appealing, once more, to the candour and good sense of those who have often heard me speak on this subject. It is notorious, that it is one half the business of grooms, who in general are the managers of their masters' stables, to stop up every crack and crevice to prevent, as they say, horses catching cold, although it cannot be admitted that their general system of treatment is but ill calculated to produce the desired effect. Looking at the matter, however, in a general point of view, as Professor Peal and Coleman observes, and speaking in the ordinary acceptation of the word, cold may be considered as a sort of open, undisguised enemy, whose attacks are constantly suspected, and whose approaches every one is prepared to repel. But, *volatile alkali is an enemy of a very different description, a foe that no one suspects, who silently and imperceptibly enters the citadel in the disguise of a friend, and thereby gradually undermines the very foundation of the edifice.* Before I conclude the subject of moon blindness, it may be necessary to notice some circumstances connected with this disease. Now in moon blindness, it mostly happens, that one eye only is attacked with inflammation, and on the disease subsiding, the other eye immediately becomes affected. Thus the disorder continues to fly from one eye to the other alternately, till a cataract takes place in both, and the horse becomes totally and incurably blind; but if the inflammation (how frequently soever it may fluctuate) continue confined to one eye only, and it ends finally in a hard, sometimes a bony cataract, the probability is, that the other eye will remain sound for many years, and perhaps during the lifetime of the animal.

The fact is so commonly known, that it has given rise to a practice at once both cruel and impolitic, that of thrusting a red hot needle or awl into the diseased eye, with a view to the total destruction of the power of vision in the organ.

But the *vehemence* of the inflammation which is excited in the diseased eye by the operation, is sometimes so prodigious, that the sound eye becomes violently inflamed from sympathy, and the entire loss of both has frequently been the consequence.

Having already declared my opinion respecting the insufficiency of the most powerful medicine to remove such dense opacities of the cornea as are called films, if some application is insisted on, a little of the eye powder, No. 2, may be rubbed into the eye once or twice a week by way of rousing up the deficient energy of the absorbents.

### ON THE DISEASES OF THE HORSE'S EYE.

In describing the diseases of the horse's eye, I shall endeavour to give some useful hints to inexperienced persons, who, though they may not have studied the anatomical structure of that delicate organ, may still possess some knowledge respecting it, yet, in difficult cases, may still be desirous of consulting a veterinary surgeon.

In addition to what I shall write respecting the examination of the horse's eye, I wish to observe, that the best situation for this purpose is a stable or barn, or or under an arch way, Mr. Coleman's favourite position; so that only a moderate light may fall upon the eye; this position being the most favourable to give a distinct view of the pupil, which in the orb is of a dark bluish colour, of an oblong form, the long diameter being in a horizontal direction, as in fig. 3, 4.

The following sketch drawn up from observations made during my residence and studies at the college, on Mr. Coleman's mode of examining the eye of the horse, will perhaps give a better idea to the young student of the form of the pupil, than can be conveyed by a lengthy description. It should be considered by every person desirous of being acquainted with the eye of the horse, that the size of the pupil varies according to the different degrees of light thrown on the eye. When the horse is placed under a stable or barn door, or under an arch-way, with his face to the light, both pupils should be exactly alike, and of the same size as in fig. 3; and as you advance the horse more into the light, both pupils contract, as in fig. 4 or 5; but if either remain unaltered in their dimensions, even if exposed to a stronger light, it shows that they are affected with the disease called gutta serena; or, if any inequality be observed in the pupils when thus examined, it may safely be inferred, that one of the eyes is diseased.

A person examining a horse's eye, must place himself against the shoulder of the horse, with his face against the side of the horse's head, and by holding the bridle or head stall in the right or left hand, on which ever side he may be standing, by waving the horse's head to and fro, the object looked looked for will be obtained.

A cataract is easily distinguished, when completely formed, by the white or pearly colour of the pupil, as represented in fig. 10 or 11; but in its more incipient stage, it is not so easily detected, but by those well acquainted with its structure, as the pupil is then only rather of a lighter colour, or more cloudy or opake than natural. A partial cataract is not by some people thought of so much importance: in this opinion, however, they deceive themselves; for I seldom or ever saw a partial cataract, or one in its incipient state, be the speck on the lens ever so small, but that one time or other blindness ensued at a more remote period. Nor do I know of any disease to which the horse is subject, that requires more candour and judgment, in a professional man, than examination of horses for gentlemen under these complaints.

A partial cataract is when the white or opake speck is but small and near the margin of the pupil, as represented in fig. 6 and 7; but if there is more than one white speck, more within the centre of the pupil, or touching the lens, as in fig. 8, 9, 10 or 11, cataract is then formed, and proves not only a serious impediment to vision, but causes total blindness.

When horses are kept at grass during the hot summer months, they are very often stung about the eyes, and considerable swellings take place. The inflammation sometimes is not of much importance, and may soon go off; if, however, the horse is taken up, and his eyes bathed with the following lotion, it may soon disappear.

℞. Acetated Ceruses - - ʒi
Distilled or plain rose water, one pint.
Tincture of Opium - - ℥iss mx. and
bathe the eye two or three times a day.

But if the opacity or outer coat should continue, and a film appear to be coming over the eye, the following eye-powder must be rubbed in once every day:

℞. Sal vitriol, or white vitriol, 12gr.
Nitre pulverized - - 5gr.
Honey or molasses, q. s. made into a thin ointment, and rubbed into the eye with a feather, or the thumb.

Inflammation of the eye is often produced from various causes; such as blows, bites, stings, in which the above salve may be used, and continued until the inflammation is abated. But when inflammation is produced from other causes, such as are pointed out in this Treatise, it will require the skill and judgment of a veterinary surgeon. This salve should not be used; stimulating remedies are very often recommended for the removal of this appearance, and are sometimes a remedy, if not applied too early; that is, before the inflammation has considerably abated. I have sometimes known this medicine do good in old horses, when the pupil is considerably dilated and muddy, as it is called. In slight inflammation of the eye I have frequently used common salt well pulverized, and rubbed in under the eye lid, and all over the conjunctiva, or that part known or called by the common farriers the white of the eye.

### DESCRIPTION

OF THE

### PLATE OF THE HORSE'S EYE.

### REPRESENTED IN A DISEASED STATE.

Fig. 1—Is a morbid dilation of the pupil of the eye. The black or dark brown substance, as represented at the margin of the pupil, is a natural appearance, though sometimes mistaken for disease.

Fig. 2—Represents the pupil dilated in a less degree, with an incipient and general opacity of the part.

Fig. 3—Represents the healthy state of the pupil in a moderate light. It should therefore be observed, that as the pupil enlarges, it approaches to a circular form, as in fig. 1 and 2; and by contracting, the diameter is lengthened, as in fig. 4 or 5.

Fig. 4—Represents the pupil, when the horse is brought out of the stable, and placed in the shade.

Fig. 5—Represents the form of the pupil when the eye is exposed to the direct rays of the sun.

Fig. 6—Represents the appearance of the pupil when the eye is affected with cataract.

Fig. 7—Represents a partial cataract; that is the appearance of white or opake spots in the pupil. In this case a considerable portion of the pupil is free from spots, particularly the centre; so that vision would not be materially injured.

Fig. 8—This represents a similar disease with some slight variations, as to the situation of the spots.

Fig. 9—Represents a more circular pupil with opacity in the centre, and on the side.

Fig. 10—Represents a contracted pupil, with considerable opacity, causing almost total blindness.

Fig. 11—Represents a pupil of the natural size with opacity in the centre, materially obstructing vision.

THE

### DISEASES OF THE HORSE'S EYE

Are by no means so numerous as in the human subject, in which some hundreds have been reckoned; but, in horses, they are very obstinate of cure.

The diseases of the eyes in horses may be referred to one or two heads; the most common disease of the horse's eye is

### INFLAMMATION OF THE CONJUNCTIVA,

COMMONLY CALLED

### MOON BLINDNESS.

It is also very frequent in the horse's eye, and takes the name of Ophthalmia. This disease attacks horses very sud-

### DIFFERENT STAGES OF CATERACT

A Muscles inserted into the Serclotic Coat

B Transparent Cornea

C The Iris

D The Pupil denly, and generally takes place in the course of twenty-four hours. It begins first in the most vascular part of the conjunctiva, and then extends to the conjunctiva covering the sclerotic coat, and from that to the transparent cornea.

### SYMPTOMS OF OPHTHALMIA.

The eye-lids drop, the tears run over the cheeks, and more pass through the nasal duct, where drops of fluid may be seen at the extremity, which never are seen in a healthy state of the eye. This is an increased secretion, and no disease of the duct; the membrane nictatins, or haw, is thrown over the eye, with the dropping of the eye-lids, in order to prevent the admission of the rays of light, which in this state of the horse's eye would irritate it, and increase the disease. But ignorant smiths and farriers, mistaking the effect, and thinking the haw to be the cause of the disease, which they call moon blindness, remove this defence, which nature has given to the animal, and say that it is too long; nevertheless it is not unusual for us to find an eye that was to-day very much diseased and inflamed, quite clear in the course of the next day. This phenomenon is owing to the power of restoration being so great, in comparison to what it is in the human subject.

Now, it would be a phenomenon likewise to find this disease in an unbroke horse, it being always found in domesticated ones; in short, it is never found in colts, or in old horses, but it takes place between five and six years of age; probably the reason is, that at this period, when the animal has ceased to grow, and has arrived at maturity, then he is much more subject to disease; as we may observe in the human subject, people inclined to consumption are more liable to have it at maturity, than at any other period. For before this time the blood was required for two purposes, for nourishment, and growth of parts. But it is now wanted for one purpose only—that of nourishment; the superfluous blood must, therefore, keep the animal in a state of plethora, and consequently subject him to inflammation in various parts.

The great cause of this disease is change of temperature, vitiated by the effluvia of the dung and urine fermenting in the stables, another by forming a poison capable of producing even glanders—before the general orders or ventilation took place by the orders of government, though his majesty's barracks, there were perhaps more blind horses to be found than in all the surrounding country besides. Unequal exercise contributes also very much to produce this inflammatory disease; for it is very common to see horses exercised and ridden very violently one day, *and for a whole week after remain at comparative rest.* We are not to consider this complaint as merely local; for when it is so, it is much easier of cure; being constitutional, and therefore also requiring constitutional remedies and treatment; but unfortunately we have not discovered a specific of this nature. The horse seldom perspires in this disease; and if he does, it is in excess, which manifestly shows that the constitution is affected, and there is also a slow lingering fever. If the animal is bled, purged, &c. the eye soon becomes clear; but at the end of five or six weeks, the other eye becomes inflamed, and gets also clear, and about the same period afterwards, the eye that was originally inflamed, now again becomes infected, and so on periodically till the patient becomes totally blind in one of them.

The degree of inflammation is various; sometimes it is so great, that the iris becomes affected, and a little deposit of lymph may be observed at its edge, and also at the edge of the little glandular bodies: this does not take place in the human subject; for though the iris is contracted, it is in consequence of sympathizing with the retina to prevent the admission of the rays of light: this deposit of lymph is most commonly at the inner angle of the iris, and at the edge of the superior glandular bodies, and is a fine indication of succeeding cataract and blindness; and as it is a deposition on the iris, it is very difficult to get rid of. The cornea is also sometimes as red as if washed with the venous blood, and neither the iris nor pupil can at all be seen, which is a sure sign of the cornea being inflamed. It frequently happens that the iris appears of a yellow colour; this, however, does not indicate any disease in it, but shows an incipient disease of the cornea, which now receives more serum into its vessels, than they can make transparent, because they are too much distended and enlarged to produce the effect; just as any coloured fluid will not appear transparent, if contained in a glass tube of an increased diameter. These circumstances will also apply to the disease when going off, as well as in its incipient state. In fact, this disease may be considered as a gouty inflammation of the eyes peculiar to the horse, being a periodical disease, and having the same appearance and affections in the horse as the gout in the human subject; for it is not of the same specific nature in diseases of the eye of man, the ox, the sheep, the ass, which so frequently resemble the horse in other things. In a number of cases, the inflammation is periodical, and blindness is sure to ensue, though not always in both eyes, from whence originated among smiths, &c. the abominable custom of putting out one eye to save the other. The most common and general termination of this specific opthalmia is in a cataract, which is an opacity of the chrystalline lens, which was before transparent; it generally becomes of a white or yellow colour, &c. and inclining to white in the circumference; sometimes the capsule of the lens becomes thickened, and even bony.

### TREATMENT.

In the human subject, an operation is performed for extracting the cataract, which is generally successful in giving sight to the patient; but in the horse it is useless, and should never be recommended, because the important functions of the chrystaline lens must be supplied by two sort of glasses, convex, to see near objects, and concave, to see distant ones. Now it is utterly impossible to employ these to be of the same advantage to the horse; and without them vision is so confused, for want of the lens, that it is much better to have the animal quite blind, for he would be continually stumbling and starting at every object, of course of little value to his owner.

The only advantage of extracting a cataract in the horse would be when the animal is turned out to graze. But besides the objection before mentioned, there are still more; for the operation is very difficult to be performed, from the retractor muscle drawing the globe into the orbit, so much so that we cannot get at the cornea; besides this part is naturally much less convex than the cornea of the human subject. Mr. Coleman has, however, performed the operation by counteracting the action of the retractor muscle, by means of a double tenaculum, which is much better than a speculum, but still without success; for, after the operation is performed, the retractor muscle still continues to draw the globe into the orbit, and the eye of course appears less than its fellow, and the aqueous humour is continually escaping and preventing the union of the divided cornea, which from the irritation produced, inflames, as well as the iris and all the other parts, and the bulk of the eye is considerably diminished. The iris sometimes gets between the divided parts of the cornea, prevents the escape of the aqueous humour, and the wound heals.

Mr. Phipps has made many improvements in performing the operation on the human subject, but without any advantage to the horse, except in case of a white cataract, or if there should be lymph behind chrystalline lens and the vitreous humour; we may, in this case, remove or extract the lens, because after this is done, the animal is still blind. Another not unfrequent disease of the horse's eye is,

### THE COLLECTION OF THE MATTER

formed by the iris, which gravitates to the depending part of the anterior chamber of the eye, and has a semi-circular appearance on account of the figure of the cornea. In this the pupil is always contracted, and if it is not soon removed, by pressing on the cornea and iris, it will produce blindness; therefore, as soon as it forms, we ought to puncture the edges of the cornea, just under the matter, with the point of a fine lancet, to allow of its escape. In performing this operation at the Veterinary College, I have repeatedly seen the aqueous humour secrete again in twenty-four hours.

The last disease of the horse's eye, which is connected with Opthalamia, is

### GUTTA SERENA, commonly, by grooms and farriers, called glass-eyes, be cause the eyes generally appear very clear and glassy. It is a much more frequent affection of the human eye than of that of the horse. The pupil is very much enlarged and dilated, in consequence of sympathizing with the nerve, which now is not capable of being stimulated by the rays of light; it generally arises from an affection of the brain, as staggers, or a blow on the head. When proceeding from either of these two causes, a cure may be performed, by bleeding, purging, blistering the top of the head, &c. and stimulating the nostrils with the vapour of vitriolic or marine acid. As to that disease of the eye, called

### WATERY EYES, it proceeds from an increased secretion of tears, which flow down the cheeks, in consequence of the lachrymal ducts not being able to carry all the superfluous quantity away, or from an obstruction of the nasal ducts, which terminates in a small hole as big as a No. 1 shot, and which may always be seen by turning up the lips of the nostril at the end of the nose.

Before I close this subject, I will give some account of the different trials made by Professor Coleman, at the Veterinary College, in order to cure, or rather to prevent, the formation of

### CATARACT OR MOON BLINDNESS, as it is called by common farriers, being, as I have before observed, a specific and gouty inflammation of the eye. My worthy instructor began with bleeding from the jugular or angular veins of the face, and at the same time employing purgatives frequently repeated, as well as diuretics, administered alternately one after the other. After which he tried all the medicines of Messrs. Phipps and Wathen, but without any degree of permanent success.

The local and surgical treatment was as follows:

1st. He ordered scarification, and to pass a seaton through the membrane conjunctiva, but without effect.

2d. He removed some of the large vessels going to the cornea, and divided them with the actual cautery, but without success.

3dly. We also applied leeches to the conjunctiva, without effect.

*Note*—I have, however, succeeded with leeches, if proper ones can be obtained.

Lastly, We have taken up both carotid arteries, which was of no avail, from the anastomosis which the vertebral arteries form with them.

Therefore, the treatment comes more within the province and judgment of a veterinary surgeon, and is confined entirely to bleeding, purging, and diuretics; fomentations of warm water, in order to diminish the irritation from the tears that run over the cheeks, with plenty of moderate and continual exercise to increase perspiration.

As to the case of watery eyes, it consists in diminishing the increased secretion of tears, by judicious bleeding and diuretics, with continual and moderate exercise. But when it is found to originate from an obstruction of the ductus-ad-nasum, a veterinary surgeon should be called to open the passage, by a proper injection of limpid lintseed, by a silver pipe of at least sufficient length to reach from the nasal duct to the inner canthers or corner of the eye. But if this should fail, the ductus-ad-nasum must be opened by a proper surgical instrument, introduced with great dexterity, from the eye to the nose.

In order to form a correct judgment of the nature of this fluid, it is only necessary to consider how provident nature has been in defending this delicate organ against danger: like a diamond of inestimable value, the eye is lodged in a strong bony box, protected in front by a covering of exquisite workmanship, and always kept moist by this fluid, and which is continually supplied by the duct at the inner canthers of the eye, in order to facilitate its movements, and thereby enable it to remove all extraneous bodies, such as dust, hay-seeds, chaff, &c. for conveying it also through the bony and grisly part of the nose, which is as large as a common goose quill, and as I have before said, terminating at the end of the nostril. Should we not admire the wisdom of Providence, when we see how wisely and mechanically, like unto a pipe, or outlet to a distillery for carrying off the waste liquor, is so mechanically contrived?

It is easy to be perceived, that the eye must constantly want moisture; but could the want of the eye generate this duct, or bore the hole by which it is discharged, if nature had not thought it necessary?* There is also another fluid which lubricate the eye, and should not go unnoticed, which is, that the eye-lids of the horse are composed of a mixture of membraneous and cartilaginous substances, which terminates in that body called the tarsus. This part is extremely vascular, and its vessels are attached to what is called the *ciliary ducts*, which secretes a fluid for the purpose of moistening and lubricating the surfaces of the tarsi, and thereby preventing such consequences as otherwise constant friction would be apt to produce. When the tarsi becomes diseased, matter, as pus, is discharged, instead of healthy liquor; in this state, excoriations of the neighbouring parts, and temporary adhesions, (similar to what we sometimes feel in our own eye-lids when glued together,) may be expected. There are two muscles which perform the offices of opening and closing the eye-lids; which are not so plentifully supplied with hair in the horse, as in the human subject; they are few and scattered, yet they fully serve to protect the eye from insects or other substances. And as the tears contain a considerable portion of salt, they would constantly produce

* Nature produces nothing in vain, but ordains every thing for a wise use and purpose; why then do the smiths reason thus, and say, that the frog is of no use in the horse's foot, they therefore cut and pare it away, and thereby do more mischief in five minutes by that destructive instrument, the butrass, than nature can restore again in six months. And in order to show the evil tendency which this abominable practice at one time produced in the British army, the officers of the regiment were absolutely obliged to pass a martial law, that every farrier who was found guilty of this crimc, was branded *on his posteriors* with a hot iron in front of his regiment. And if some of the sons of Erin, at the south end of the town, were to have this operation performed on them a few times, I am confident there would not be so many lame horses in Philadelphia as there now are; for I have have been witness to the abominable practice, and stood by and seen them deprive the frog of large slices, which I am confident it was not in the power of nature to replace in as many months.

irritation on the conjunctiva of the eye, did not this membrane secrete a mucus to defend it from such an effect.

The lachrymal gland, which is situated in the outer and superior part of the eye, has five, and sometimes six, excretory ducts, which, as before observed, convey the tears over the surface of the eye, by the puncta lachrymalia, and are hence by the ductus-ad-nasam conveyed to the nose.

This puncta in the horse is much larger than in the human subject. The horse having no *lachrymal sac*, the tears therefore are designed not only to preserve the transparency of the eye, but to prevent any ill consequence which might arise from friction between the cornea palpebræ. The lachrymal gland of the horse differs also from that of the human subject in not possessing a voluntary power, subject to the emotion of the mind; and this gland in the horse's eye is frequently diseased, and its action morbidly increased, as in piphora or watery eyes: this seldom occurs in the horse, but when it does, it is occasioned by inflammation, and must be removed by the means before pointed out.

### AN EPITOME

OF

### QUESTIONS AND ANSWERS

DRAWN TOGETHER WITH A VIEW OF CONVEYING

### INSTRUCTION.

For the use of the Medical Students and others, desirous of

### STUDYING THE DISEASES,

OBTAINING A KNOWLEDGE OF THE FUNCTION, USE, AND APPENDAGES OF

### THE HORSE'S EYE.

---

### 1. What is the Eye?

It is the immediate organ of vision.

---

### 2. How many coats on the Horse's Eye?

Four, viz.—The Conjunctiva, the Sclerotic, the Choride, and the Retina.

---

### 3. What is the Conjunctiva?

A thin transparent vascular membrane lining the lids, and covering the opake cornea and membrane nictitans. The part over the cornea carries only serous blood, except in inflammation.

---

### 4. What is the sclerotic coat?

A tendonous opake body, is very little vascular, has very few nerves, and contain the humours of the eye.

---

### 5. What is the choride coat?

It is a vascular tunic, attached to the posterior and inner surface of the clerotic coat; its inferior and posterior part is covered by nigrum pigmentum, and its anterior and superior part in the horse is green.

---

### 6. What is the retina?

A thin transparent vascular membrane, being the posterior part of the eye, anterior to the choride coat, and posterior to the viterous humour. But Mr. Bell divides it into two coats or lamina, the anterior of which is called the tunica vasculosa retina, or the central artery ramnifies the substance of the nerve.

---

### 7. What is the use of the Conjunctiva?

In the first place it prevents any extraneous bodies getting into the posterior part of the eye, and there are small glands placed in the posterior surface called glandular sebaca, which secretes a mucus to defend it, the eye from irritation of the tears which escapes at small openings all over the transparent covering to the transparent cornea.

### 8. What is the transparent cornea?

It is a dense transparent body, a little vascular, (except in disease) few nerves, situated at the interior part of the eye under the conjunctiva; it is fitted into the opake cornea or sclerotica, as a watch glass is fitted into its case.

---

### 9. What is the use of the choride coat?

Its principal use is, that the black parts absorb the rays of light, that enters the eye from above, and the green part reflects them back again, by which means they strike the retina twice, and gives as it were a stronger vision, by making a stronger impression on the optic nerve.

---

### 10. What is the use of the sclerotic coat?

It is to contain the humours of the eye, and for the attachment of the seven muscles; its density prevents any enlargement of the parts, its being inelastic.

---

### 11. What is the use of the retina?

It is the immediate organ of vision. It receives impressions, and conveys the same to the brain to perfect vision.

---

### 12. How many humours has the eye?

Three; the aqueous, vitreous, and the chrystalline lens.

### 13 What is the aqueous humour?

A thin transparent watery fluid occupying the anterior and posterior chamber of the eye; it is convex anteriorly, and adapts itself to the convexity of the transparent cornea, and concave posterior adapting itself to the anterior convexity of the chrystalline lens.

---

### 14. What parts the aqueous humour?

The iris.

---

### 15. What is the crystalline lens?

A transparent lenteform body, hard in its centre, but less so in its circumference; it is contained in its bag or capsule, which is a continuation of the tunica vetera, and is attached to the cellular processes or ligaments; its anterior surface lies in contact with the aqueous humour, close to, but not touching the iris. The posterior surface is imbedded in the vitreous humour.

---

### 16. What is the viterous humour?

That pellucid body which occupies all the posterior parts of the eyes that is not occupied by the choride coat and retina posterior to the crystalline lens; it is concave posterior, and slightly convex anterior; it is contained in circumscribed cells or cavities, which are beautifully transparent and vascular, from which the fluids are secreted.

---

### 17. What is the liquor margani?

A small drop of transparent fluid contained between the crystalline lens and its capsule.

Its use, is to prevent friction between them; it is secreted by the exhalent vessels of the external surface of the lens, or the internal surface, or perhaps by both.

---

### 18. What is the use of the aqueous humour?

It gives a convex shape to the eye anteriorly by pressing out the transparent cornea; it also assists in refracting the rays of light, and allows the iris to perform its functions by floating in it.

---

### 19. What is the use of the crystalline lens?

Its use is very important; for without it, or substitutes for it, the eye would be of little use. Its principal use is, in bringing all the rays of light that pass into one focus on the retina, by whose aid the objects are pointed in it, to be conveyed to the brain. It is also a transparent lentiform body, contained in a capsule of its own, and is imbedded posteriorly in the vitreous humour, being surrounded anteriorly by an opake matter, termed the celliary processes, and between the capsule and its lens is the liquor margani. Mr. Coleman accounts for the eye adapting itself to near or distant objects by acting as a telescope and microscope to different densities of the middle edges of the lens. He accounts for it in this way: That in viewing a near object, as a fly, the iris dilates, the pupil becomes small, and compels all the rays of light to pass through its centre or most dense part. Whilst viewing hills, or any object at a distance, the iris contracts, consequently the pupil becomes as large as possible to take in all the rays of light through the circumference or less dense part of the lens. This action, by Sir Everard Home, was accounted for by the action of the muscles in altering the convexity of the transparent cornea.

### 20. What is the iris?

It is an oblong body attached to the tunica sclerotica about a line's breadth posterior to the junction of the transparent cornea with the opake cornea or sclerotic coat, and is said to be the only part that perform the office of a muscle and a gland. It is kept in its situation by, and floats in the aqueous humour. It is composed of two sets of fibres, the one radiated, and the other circular, or as other spincta muscles.

### 21. What is the use of the iris?

It acts as an internal eye-lid in shutting out the superior rays of light, and it is instrumental in altering the eye from a near to a distant object, and secretes the aqueous humour.

### 22. What are the cellary processes?

They are a continuation or reflection of the choride coat, being thrown into folds, and covered by nigrum pigmentum on both sides, and occupies all that part between the cornea and crystalline lens.

### 23. What its use?

To secrete the aqueous humour, and prevent the rays of light from passing through the vitreous humour without first passing through the lens, to prevent confusion of vision.

---

### 24. What is the nigrum pigmentum?

A black or other coloured pigment covering part of the choride coat, iris, and cellary processes; it is classed by some veterinarians with the rete-mucosum, or middle skin, it giving colour to the iris, &c. whence blue, black eyes, &c. where the hair is light, the eyes are so, and vice versa.

---

### 25. What is its use?

Its use is to absorb the rays of light that are not wanted for vision. It is secreted by the exhalent vessels of the parts which it covers. There is also in the eye of the horse three or four glandular bodies placed at the superior edge of the iris, which is covered by ligaments, and which, when the pupil is dilated, totally disappears; but when the pupil contracts, or becomes small, these bodies appear, and their use is very obvious, which is in absorbing and shutting out some of the rays of light when too strong.

---

### ON THE DISEASES OF THE HORSE'S EYE.

### 26. What diseases are the horse's eye subject to?

Strictly speaking only two, which are inflammation and its consequences, and which are ophthalmia, cataract, &c. and gutta serena or paralisis of the optic nerve.

### 27. What is the cause of inflammation?

Change of temperature, foul air, foul stabling, and unequal exercise also contribute to produce this disease.

---

### 28. What are the symptoms?

The eye-lids drop, the tears flow over the cheek, the membrane nictitans (called the hooks) is generally drawn over the eye, to prevent irritation from the rays of light.

---

### 29. How would you treat the disease?

As for local applications they are seldom of any avail, except in the most incipient stage of the disease. Mr. Coleman mentions numerous experiments, which were of little or no effect. He has opened the temporal veins, taken up the carotid, maxillary, and temporal arteries; in fine, he has tried most of the specifics of the human subject of Messrs. Phipps and Walthen, oculists, without any permanent good. This disease the common farrier calls moon blindness. It has an inflammation very like the gout, an eye being diseased at a time, and that eye will frequently get clear in the course of one night, and the other become affected. If the inflammation proceed from a blow on the head, the usual prescriptives here laid down will generally effect a cure; but if it is constitutional, there is seldom any permanent relief. You will generally find that it attacks horses about five or six years of age, and seldom after eight or nine; before five or six years old, the blood is wanted for two purposes, namely, for the natural secretion of the growth of the animal, after which time it is wanted but for one purpose; consequently, the system is more plethoric, and likely to take a more inflamed action. I do not mean to say that any age is exempt from it; this disease puts on different appearances; sometimes the cornea is red, sometimes yellow, and sometimes of an opake white, and inflammation may go on without redness of the part, as in the chrystalline lens; sometimes, to a superficial observer, it would not appear at all inflamed; it generally attacks the horse in the night, and gets better in the day time. A proof that foul air aggravates the disease is, that in the cavalry, ten horses lost their eyes from standing on litter all day long; and now these causes are removed, from 125 to 150 horses are saved in every regiment.

---

### 30. What is a cataract?

An opacity of the chrystalline lens or its capsule, preventing the rays of light passing to the retina.

---

### 31. How would you ascertain this disease?

I should place the horse with his head, under an arch, or out of the stable door, then placing myself against his shoulders. Should a small spot appear in the middle of the lens, I would pronounce cataract. But if not perfectly satisfied, I should get a candle, and hold in the front, and then place myself posterior to the horse's eye, to see more distinctly.

---

### 32. What is gutta serena?

It is a parallisis of the optic nerve.

### 33. What are its symptoms?

A total loss of power of the optic nerve, so that light makes no impression on it, the pupil being insensibly dilated, and the eye at the same time appearing perfectly clear and bright. It is termed by the common farriers glass eyes.

---

### 34. How would you treat this disease?

I would bleed, give physic, and apply stimulants to the eye, and blister the head.

---

### 35. What are wall eyes?

Nothing more than a light coloured pigment covering the iris.

---

### 36. What divides the eye into two chambers?

The iris.

---

### 37. Which is the largest humour of the eye?

The viterous.

---

### 38. Which is the thickest humour of the eye?

The crystalline lens.

### 39. What is the use of the eye?

It is to receive, refract, and unite the rays of light, and paint the object upside down on the retina.

### A TABLE

### OF THE

### MUSCLES OF THE HORSE'S EYE.

| NAMES. | ORIGIN. | INSERTION. | USE. |
| --- | --- | --- | --- |
| Orbicularis palbebræ or sphincter. | From all round orbit from the os frontis—ungus, malar and temporal bones. | Into the skin of the eye-lids, all round being a sphincta muscle. | To shut the eye. |
| Levator palbebræ. | From the posterior part of the orbit. | Into the eye-lids. | To open the eye-lids in opposition to the above. |
| The short oblique. | From the anterior and inferior part of the orbit, from the orbicularis near the nasal duct. | And is inserted obliquely into the sclerotic coat. | To draw the eye forward, as is seen by the starting when the animal is alarmed. |
| Long oblique trochlearis. | From the posterior part of the orbit a little above the short oblique from the sphenoid bone. | Passes through a trocherais, or pully turns round, and is inserted into the inner and upper part of sclerotic coats. | Draws the eye forward and towards the body, and passes through it, as in the os frontis. |

| | | | |
|---|---|---|---|
| The adductor oculi. | From the posterior part of the orbit. | Into the inner part of the sclerotic coat. | To draw the eye towards the head. |
| The depressor oculi. | From the posterior of the orbit below the adductor. | In the anterior and inferior part of the sclerotic coat. | To derpress the eye when looking towards the ground. |
| The abductor oculi. | From the posterior part of the eye. | Into the anterior and exterior part of the sclerotic coat. | To draw the eye from the body. |
| The levator oculi. | From the superior part of the orbit above the last mentioned muscle. | Into the anterior and superior part of the sclerotic coat. | To draw the eye upwards. |
| The retractor oculi. | From the posterior part of the orbit, and surround the optic nerve. | Into the whole part of the sclerotic coat. | To draw the eye into the orbit. |

### THE MUSCLES AND APPENDAGES OF THE HORSE'S EYE.

### 1. The lachrymal gland

Is placed in the superior and outer canthus or corner of the eye, under the orbitary process of the os frontis.

### Its use

Is to secrete the tears.

---

### 2. The cellular ducts

Pierce the conjunctiva at its superior part, and convey the tears to the anterior part of the eye.

---

### 3. The tears

Are secreted by the lachrymal gland, most during day. The use of the tears are to wash the anterior part of the eye, assist in removing extraneous bodies, and to keep the eye transparent.

---

### 4. The conjunctiva

This is a vascular membrane lining the eye-lids, and is reflected over all that part we touch with the finger in the living animal; it is very vascular in that part lining the lids, but less so covering the opake cornea, and still less so covering the transparent cornea.

### Its use

Is to prevent extraneous bodies getting into the eye, and that part lining the lids secretes a mucus to prevent the tears excoriating the eye, which mucus is conveyed over the eye by the motion of the lids.

---

### 5. Puncta lachrymalis

Are two; the inner canthus or corner of the eye, within the conjunctiva, one on the upper, and one on the lower eye-lid, and when joined are large or larger than the nasal duct.

Their use is to carry the tears to the nasal duct.

---

### 6. The ductus ad nasum

Perforates the os unguis, and is in part bony, and in part membraneous, and terminates nearly at the extremity of the nose.

### Its use

Is to carry off the tears.

---

### 7. Caruncula lachrymalis

Is a small black body at the inner canthus of the eye, between the two punctures, and is not entirely covered by conjunctiva.

### Its use

Is to direct the tears between the two punctures.

---

### 8. The transparent cornea

Is oblong in form, and is composed of dense transparent materials, being a sort of strata, or layer upon layer, is convex anteriorly; it is also imbedded in the opake cornea like the glass to a watch case, and is broad at the inner canthus.

### Its use

Is to assist by its convexity and density, by breaking down the rays of light, and its connexion with the opake corner, assists in retaining the aqueous humour in its situation.

*Note*—It has absorbents and nerves.

---

### 9. The opake cornea or sclerotica

Is the outer coat of the eye, and is attached by cellular membranes to the posterior appendages, as muscles, &c. it is opake and inelastic, and is perforated by the optic nerve.

### Its use

Is to contain the viterous humour, and to protect the posterior part of the eye. It has but few blood vessels, absorbents or nerves.

---

### 10. The chorides

Is a black reticular membrane composed of two layers, and its lining the sclerotica, and is covered in part by a black pigment, and in part by a variegated coat. The variegated coat is the superior and largest in proportion. *Note*, by taking an ox's eye, and cutting it transversely across, and letting out the humours and the lens, the two variegated colours, black and green, are beautifully exemplified.

### Its use

Affords a surface for the expansure of the optic nerve, and the black inferior pigment called the nigrum pigmentum absorbs the superfluous rays of light, while its variegated superior covering reflects them back again, and thereby striking the optic nerve twice, and giving a double effect to the organ of vision. It is very vascular, and has numerous absorbents and nerves.

---

### 11. The retina

Is an expansion of the optic nerve on the anterior surface of the choride coat, having a web-like membraneous appearance.

### Its use

Is to convey the sensation of light to the brain.

---

### 12. The aqueous humour

Is a watery fluid contained in the anterior and posterior chamber of the eye, and is supposed to be secreted by the cellular processes of the iris.

### Its use

Is to keep the transparent cornea convex, to reflect the rays of light, and enable the iris to perform its functions, by affording a space for its contraction and dilation.

---

### 13. The anterior chamber

Is all that space between the posterior part of the transparent cornea, and the anterior part of the iris, and is filled with aqueous humour.

---

### 14. The posterior chamber

Is all that space between the posterior surface of the iris, and the anterior surface of the chrystalline lens, and is filled with aqueous humour.

---

### 15. The chrystalline lens

Is a transparent lentiform body, contained in a body or capsule of its own, and is imbedded posteriorly in the vitreous humour, being surrounded anteriorly by an opake matter, termed the cellulary processes, and between the capsule and its lens is the liquor morgiani to prevent adhesion; it is convex on each side, but most in its posterior part; its centre posteriorly being the hardest, and more convex than the other. It is broader and flatter anteriorly, than it is posteriorly.

---

### Its use

Is to refract, break, or bend the rays of light, and bring them into one focal point on the retina; its hard broad centre acting as a microscope, and its flat softer margin performing the office of a telescope. Its microscopical powers to obtain a knowledge of near objects, and its telescropical powers to obtain a knowledge of distant objects. It is supposed to compose a number of very minute tubes, containing white blood, arteries, nerves and absorbents, and is supposed by Astley, Cooper, and Professor Coleman, to be the seat of cataract.

---

### 16. The cellulary processes

Are a continuation or reflection of the choride coat being thrown into folds, and covered by nigrum pigmentum on both sides, and occupies all that space between the cornea and chrystalline lens, and lays in contact with the outer portion of the chrystalline lens.

---

### Their use

Is to secrete the aqueous humour, and prevent the rays of light from passing through the vitreous humour, without first passing through the chrystalline lens, to prevent confusion of vision.

---

### 17. The cellulary ligament

Is seen and situated just before the chorides gives off the cellulary processes. The outer lamina of the chorides being formerly fixed to the sclerotic coat by a sort of ligamentous circle, which is termed the cellulary ligament.

---

### Its use

Is to connect the outer lamina of the choride coat more firmly to the sclerotic.

### 18. The vitreous humour

Occupies the whole back of the eye, posterior to the chrystalline lens, so that the posterior part of the lens is imbedded in its soft and delicate surface, and is admirably adapted to lay in contact with the retina. It is composed of a number of circumscribed cells, containing their watery fluid within a tunic, but it has one membrane, containing or enveloping the whole.

---

### Its use

Is to expand the bulb of the eye, posteriorly to lay in contact with the retina, and to bend or refract the rays of light passing through the optic nerve.

---

### 19. The Iris

Is a muscular body, having two orders of muscular fibres entering into its composition: the one orbicular, and the other radiated, and is covered with nigrum pigmentum on both sides; it is attached to the transparent cornea, one linc's breadth anterior to its junction with the sclerotic coat.

---

### Its use

It forms the colouring matter of the eye, assists in secreting the aqueous humour, and by its power of contraction and dilation regulates the quantity of light passing through the crystalline lens. The blood vessels are curious, one artery going round its circumference, and another round its centre. There are also numerous small arteries connecting the two like the spokes of a wheel, so that in structure it resembles a coach wheel with its spokes. At the internal margin of the iris are seen some little glandular bodies, three or four superior, and sometimes one or two inferior, but the superior are the largest, and they are covered by nigrum pigmentum.

---

### Their use

Are to assist the iris in regulating the rays, when too strong.

---

### 20. The membrane nictitans

Is a cartilaginous body placed at the inner canthus of the eye, having one part anterior, and one part posterior to the transparent cornea; it is in part covered, and its edges or two sides fastened by conjunctiva, and is edged with black. It is attached by cellular membrane to the posterior part of the eye, and may be considered as an appendage to the retractor muscle, its action being dependant on that. It is entirely concave, externally convex; and, when under inflammation, it projects, particularly in lock jaw, owing to the retractor muscle drawing the eye inward, to avoid the irritation produced by the rays of light. This is what the *ignorant part of the community* take for the hooks in lock jaw.

---

### Its use

Is to defend the eye from injury, and to wipe off any extraneous bodies, such as sand, insects, hay-seed, and to assist in cleansing the eye with the tears, as a sponge does with water.

### 21. How many muscles has the horse's eye.

Seven; the elevator, the depressor, the abductor, the adductor, (long and short oblique,) being the oblique externus and oblique internus, and the retractor oculi, which acts in the eye when under inflammation, and which grooms and smiths call the hooks.

---

### 22. Give their origin and insertion.

The four recti or straight muscles, which are elevator oculi, draws the eye upwards. The depressor oculi draws it downward, as when in the act of grazing; the adductor draws the eye towards the forehead, and the adductor draws it towards the temple; they all arise from the bottom of the orbit, and are inserted in their tendons into the clerotic coat about half an inch posterior to its junction with the transpa rent cornea. The oblique externus, trochelaris or oblique muscle arises from the back part of the orbit, and passes through a ligamentous trochelaris or pully, at the superior part of the orbit, and is inserted into the anterior and superior part of the orbit, and is inserted into the anterior and superior part of the clerotic coat, and draws the globe outward and inward. The oblique internus or obliqueous brevis, arises from the inferior and anterior part of the orbit near the ductus-ad-nasam, and is inserted into the inferior and superior part of the clerotic coat. It serves also to draw the eye inward, and Mr. Coleman supposes, that when these muscles act together, that they rotate the eye. The retractor oculi arises from the bottom of the orbit, and is inserted all round the posterior part of the clerotic coat. But Dr. Munro supposes, that its use was to support the eye whilst the animal was grazing, without the assistance of the retractor oculi.

But this, Mr. Coleman says, cannot be the case, as other animals have it which do not graze, as dogs, wolves, bears, lions, tigers, &c. which are carnivorous animals. It is therefore evident, that the principal use of the muscle is, to draw the membrane nictitans, or curtain of the eye, called by some the haw or hooks, into the orbit, when the eye is suffering under inflammation, to defend it from the superfluous rays of light.

## Figures and Tables

**Figure f1:**